# Amblyopia is associated with impaired balance in 3–6-year-old children in China

**DOI:** 10.3389/fnins.2022.993826

**Published:** 2022-09-23

**Authors:** Taylor A. Brin, Zixuan Xu, Yusong Zhou, Lei Feng, Jinrong Li, Benjamin Thompson

**Affiliations:** ^1^School of Optometry and Vision Science, University of Waterloo, Waterloo, ON, Canada; ^2^State Key Laboratory of Ophthalmology, Zhongshan Ophthalmic Center, Sun Yat-sen University, Guangzhou, China; ^3^Center for Eye and Vision Research Limited, Hong Kong, Hong Kong SAR, China; ^4^Liggins Institute, University of Auckland, Auckland, New Zealand

**Keywords:** amblyopia, strabismus, motor function, binocular vision, stereopsis

## Abstract

**Purpose:**

School-age children in China have more advanced motor development than their North American counterparts. This is likely due to cultural differences in children’s regular motor activities. It is unknown whether the motor function impairments associated with binocular visual disorders (BVDs) such as amblyopia in children raised in North America exist for children raised in China.

**Design:**

Prospective case-control study.

**Methods:**

A major tertiary eye hospital in China tested children aged 3 to <7 (*n* = 63) belonging to three groups: anisometropic or strabismic amblyopia (*n* = 22), anisometropia or strabismus without amblyopia (*n* = 20), or controls (*n* = 21). The main outcome measure was motor function scores (Movement Assessment Battery for Children 2nd edition).

**Results:**

Balance scores varied significantly across groups (*F*_2,61_ = 4.2, *p* = 0.02) with the amblyopia group (mean ± SD: 12.5 ± 3.0) exhibiting significantly poorer scores than controls (14.8 ± 2.3). The no-amblyopia BVD group (12.8 ± 3.1) did not differ significantly from the other groups. Manual dexterity, catching and throwing and total scores did not vary significantly across the three groups. A separate pre-planned comparison of only the amblyopia and control groups revealed significantly poorer total motor scores in the amblyopia group (10.1 ± 3.2) vs. controls (12 ± 2.4). A linear regression model was unable to significantly predict associations between total motor score and binocular function score (standardized β = −0.09, 95%, *p* = 0.7), amblyopia etiology (standardized β = 0.14, 95%, *p* = 0.4), or inter-ocular acuity difference (standardized β = −0.18, 95%, *p* = 0.4), in the amblyopia group.

**Conclusion:**

Amblyopia is associated with motor function impairment in children raised in China. Motor deficits that may impact everyday activities have been observed in patients with amblyopia across multiple cultures.

## Introduction

It is well established that visuomotor skills are impaired in European and North American samples of patients with amblyopia (Grant and Moseley, 2011; Grant et al., 2014; Birch et al., 2019a). Specifically, when compared to controls, individuals with amblyopia exhibited reduced accuracy, speed, or both on everyday visuomotor tasks such as grasping objects (Grant and Moseley, 2011). In addition, children with strabismus or anisometropia (with or without amblyopia) were 3–6 times more likely than controls to have a total score below the 15th percentile on the Movement Assessment Battery for Children 2nd edition (MABC-2; a standardized pediatric test of gross and fine motor function) (Kelly et al., 2020), indicating the possibility of a clinically important motor function disorder.

Motor development is influenced by culture. For example, children raised in China had a significantly better fine and gross motor function than their peers in Europe and North America (Pang and Fong, 2009). For the MABC-1 test in particular, children in China aged 3–10 had significantly higher manual dexterity scores than children in the UK (Ke et al., 2020). In Hong Kong, children had better manual dexterity and balance scores than children in North America (Chow et al., 2001). This effect was most pronounced at younger ages suggesting accelerated motor development in Hong Kong children. Possible explanations include the use of chopsticks by the age of 2 and attendance of mandatory, highly academically oriented preschool programs before the age of 7 (Chow et al., 2001). Because all previous studies of motor function and amblyopia have assessed European or North American samples of patients, it is currently unknown whether cultural influences alter the association between amblyopia and motor dysfunction. This is an important knowledge gap because motor function deficits are being recognized as a fundamental symptom of amblyopia that reduces self-esteem and self-perception of physical competence (Kelly et al., 2015; Birch et al., 2019b). In addition, motor function is now being included as an outcome measure in tests of new amblyopia treatments (Webber et al., 2016). Here, we tested groups of children in China with binocular visual disorders (BVDs) including amblyopia, strabismus, or anisometropia without amblyopia, and controls to assess the associations between BVDs and motor function in this cultural group.

We included a no-amblyopia BVD group because Kelly et al. (2020) observed a different pattern of motor deficits for patients with no-amblyopia BVD compared to those with amblyopia. The no-amblyopia BVD group exhibited significantly worse manual dexterity scores on the MABC-2 compared to controls. However, patients with amblyopia were significantly worse than controls for all components of the test (manual dexterity, catching and throwing, balance, and total score).

It was hypothesized that the pattern of motor deficits seen in other cultures would also occur in children raised in China. The protective factors that cultural differences in motor development may grant are likely not enough to mitigate the predicted effect of amblyopia on motor development. It was also predicted that stereopsis would be associated with motor function deficits. Stereopsis is crucial for gross motor skills such as catching a ball (Mazyn et al., 2007) and gait during obstacle navigation (Buckley et al., 2010), as well as performing fine motor tasks such as the Purdue pegboard and bead-threading (O’Connor et al., 2010). In amblyopia, Suttle et al. (2011) observed an association between poorer stereopsis and slower, less accurate reaching and grasping. Grant et al. (2014) also observed that individuals with nil stereopsis exhibited more frequent reaching and grasping errors than those with measurable stereopsis on the Titmus fly test. However, multiple studies have observed no association between stereopsis and motor function deficits in patients with amblyopia and binocular vision disorders (Webber et al., 2008; Zipori et al., 2018; Ibrahimi et al., 2021) suggesting that factors other than stereopsis may influence motor performance in these groups.

The association between amblyopia and motor function deficits is important because the real-world consequences of impaired motor function are far-reaching and impact on overall well-being. Open questions include the relative importance of specific amblyopia symptoms such as visual acuity loss and impaired stereopsis for the development of motor dysfunction. The goal of this study was to study the associations between BVDs with and without amblyopia and motor function deficits in children raised in China and to assess whether stereopsis was associated with motor deficits.

## Materials and methods

### Participants

Ethics was approved by the Zhongshan Ophthalmic Centre Ethics Review Board and the University of Waterloo Ethics Review Board prior to the start of the study and is in compliance with the declaration of Helsinki. All participants and their guardians went through the informed consent process. Participants were tested at Zhongshan Ophthalmic Centre, a major tertiary eye hospital in Guangzhou, China. Children aged 3 to <7 years old were recruited across three different groups: amblyopia, no-amblyopia BVD, and control. Evaluators were masked to the group each patient belonged to during the entire testing process. Participants in the amblyopia group had a diagnosis of anisometropic, strabismic, or mixed amblyopia, best-corrected visual acuity (BCVA) of 0.3–1.0 logMAR (inclusive) in the amblyopic eye, and age-dependent normal visual acuity in the fellow eye (0.3 logMAR for 3–4 years of age, 0.2 logMAR for 5 years of age). The interocular difference in visual acuity was 0.3 logMAR or greater. Anisometropia was defined as an interocular difference in spherical equivalent refraction >1 diopter. Participants in the no-amblyopia BVD group had anisometropia or strabismus without amblyopia (weaker eye BCVA better than 0.3 logMAR, interocular acuity difference less than 0.3 logMAR). Control group participants had normal stereopsis (400” or better for 3 years of age, 200” or better for 4 to <7 years of age) (Birch et al., 2008), no previous history of amblyopia or BVD and age normal visual acuity in both eyes. Exclusion criteria for all groups were the presence of an eye disease or visual disorder other than amblyopia, strabismus, or anisometropia, premature birth (<32 weeks of gestation), systemic disease, developmental delay, and vestibular disorder. Eligible participants completed a single 2-h visit for data collection. The study protocol was approved by the Zhongshan Ophthalmic Centre Institutional Review Board. Written parental consent was provided for all study participants.

### Motor function assessment

The MABC-2 is a collection of age-normed tests aimed to assess motor skill dysfunction in children. The MABC-2 tasks were designed to resemble everyday activities and are listed in Table 1. The scoring system was developed using normative data obtained from over a thousand children. The tests vary depending on the age of the child being assessed, across three separate age bands. The 3–6-year age band tests were used for this study (Table 1). Participants were assigned an age-standardized score (1–19) and percentile rank (1–99) for each test based on their performance. Higher scores indicate better performance. A score under the 15th percentile in any category indicates that the child is at risk of having a motor function disorder.

**TABLE 1:** Individual tasks from each category of the MABC-2 for 3–6-year-old children.

Category	Task	Scoring dimension
Manual dexterity 1	Posting coins (preferred hand); posting coins (non-preferred hand)	Time to completion (seconds)
Manual dexterity 2	Threading beads (bimanual)	Time to completion (seconds)
Manual dexterity 3	Drawing a trail within guidelines (preferred hand)	Number of errors
Aiming and catching – catching	Catching a beanbag	Number of successful catches (out of 10)
Aiming and catching – throwing	Throwing a beanbag onto a target mat	Number of successful throws onto the target (out of 10)
Balance – static balance	Balancing on preferred leg; balancing on non-preferred leg	Time spent balancing (seconds) (max score = 30 s)
Balance – dynamic balance 1	Walking along a straight line with heels raised	Number of steps without a mistake (max score = 15)
Balance – dynamic balance 2	Jumping from one mat to another	Number of hops without a mistake (max score = 5)

### Visual function tests

The PEDIG ATS-HOTV system was used to test visual acuity (Moke et al., 2001). Interocular suppression was measured with the Worth 4 Dot test at far (6 m) and near (33 cm) distances. The Randot^®^ Preschool Stereoacuity Test (RPST) was used to measure stereopsis.

### Analysis

Statistical analyses were conducted using JASP (JASP Team 2020, Version 0.14.1). Nil stereopsis was assigned an arbitrary value of 10,000 arc sec (O’Connor et al., 2010). Each participant was also assigned a binocular function (BF) score based on their stereopsis and Worth 4 Dot test results (Webber et al., 2019). Those with no measurable stereopsis who also could not fuse during the Worth 4 Dot test were given a score of 5. Those with no measurable stereopsis, but the ability to fuse during the Worth 4 Dot test were given a score of 4. The scores of those with measurable stereopsis are calculated by taking the log of their stereoacuity (in sec of arc). The result is a range of scores to 5 where lower scores indicate better BF.

Between-group differences (control vs. no-amblyopia BVD vs. amblyopia) in total MABC-2 standard scores and standard scores for each MABC-2 subscale (manual dexterity, aiming and catching, and balance) were assessed separately using one-way ANOVAs. Significant main effects were explored using *post hoc* pairwise comparisons, with all *p*-values corrected using Tukey HSD to account for multiple comparisons. A pre-planned analysis comparing only the control and amblyopia groups was also conducted. Bonferroni corrected *t*-tests were used to compare each of the eight subsections of the MABC-2.

A linear regression was used to quantify the association between MABC-2 total standard scores in the amblyopia group with the following covariates: stereopsis (BF score), amblyopia type (strabismus/no strabismus), and interocular acuity difference. Several exploratory analyses replacing total motor function score with balance, catching and throwing or manual dexterity were also run.

## Results

### Patient characteristics

A total of 73 participants were screened. Ten participants were excluded for not meeting the study inclusion criteria. The composition of the remaining 63 participants was: control *n* = 21, no-amblyopia BVD *n* = 20, and amblyopia *n* = 22 (Table 2).

**TABLE 2:** Clinical characteristics of participants separated by group.

	Control (*N* = 21)	BVD (*N* = 20)	Amblyopia (*N* = 22)	*P*-value
**Etiology**				
Anisometropia, *N* (%)	0 (0%)	7 (35%)	11 (50%)	<0.001*
Strabismus, *N* (%)	0 (0%)	13 (65%)	11 (50%)	<0.001*
Mean age (months)	58.9 (SD = 11)	65.9 (SD = 8.7)	65.9 (SD = 9.4)	0.02*
Female, *N* (%)	8 (38%)	10 (50%)	9 (41%)	0.9
Abnormal Worth 4 Dot (near or far), *N* (%)	3 (14%)	8 (40%)	21 (95%)	<0.001*
Median near stereopsis (arc sec), (interquartile range)	32 (25–160)	100 (47.5–200)	10,000 (2,800–10,000)	<0.001*
Mean BF score	1.7 (SD = 0.5)	2.1 (SD = 0.8)	3.9 (SD = 1.0)	<0.001*
Nil near stereopsis, *N* (%)	0 (0%)	1 (5%)	16 (72%)	<0.001*
**Amblyopic/worse eye visual acuity (logMAR), *N* (%)**				
≤0.1	18 (86%)	14 (66%)	0 (0%)	–
0.2 to <0.3	3 (14%)	5 (24%)	0 (0%)	–
0.3 to <0.4	0 (0%)	1 (5%)	5 (23%)	–
0.4 to <0.5	0 (0%)	0 (0%)	6 (27%)	–
0.5 to <0.6	0 (0%)	0 (0%)	6 (27%)	–
0.6 to <0.7	0 (0%)	0 (0%)	2 (9%)	–
≥0.7	0 (0%)	0 (0%)	3 (14%)	–
Mean visual acuity of amblyopic/worse eye (logMAR)	0.07 (SD = 0.07)	0.10 (SD = 0.11)	0.47 (SD = 0.14)	<0.001*
Median inter-ocular VA difference (logMAR) (interquartile range)	0 (0–0)	0 (0–0.1)	0.3 (0.15–0.48)	<0.001*

The *p*-value indicates whether there was a main effect of group within a one-way ANOVA or a Chi-square test.

### Motor function: Standard scores

A summary of the standard scores obtained for each MABC-2 component is shown in Table 3. MABC-2 total (*F*_2,60_ = 2.7, *p* = 0.08), manual dexterity (*F*_2,60_ = 1.4, *p* = 0.23), and aiming and catching (*F*_2,60_ = 1.4, *p* = 0.3) standard scores did not vary significantly across the three groups. However, a significant main effect of group was present for the balance standard scores (*F*_2,60_ = 4.3, *p* = 0.02) whereby patients with amblyopia had significantly worse scores than controls (*p* = 0.02).

**TABLE 3:** Mean standard scores for each component of the MABC-2.

	Mean standard score in each task (SD)											
	Manual dexterity				Aiming and catching			Balance				Total
	Coins	Beads	Trail	Total	Catch	Throw	Total	One-foot	Heels raised	Jumping	Total	Total
Controls	9.5 (2.4)	9.1 (3.2)	9.8 (3.1)	9.8 (2.3)	9.8 (3.4)	11.6 (2.2)	11.2 (2.8)	12.8 (2.5)	12.4 (0.7)	11.9 (0.3)	14.8 (2.3)	12 (2.4)
BVD	9.5 (3.1)	10.4 (3.4)	6.8 (4.2)	8.6 (3.4)	9.2 (2.2)	11.4 (3.5)	10.8 (2.4)	12.2 (2.0)	11.0 (3.0)	11.9 (0.4)	12.8 (3.1)	10.6 (2.8)
Amblyopia	7.5 (2.7)*	8.9 (4.1)	8.1 (4.2)	8.3 (3.6)	8.0 (2.7)	11.1 (3.4)	10.0 (2.4)	11.6 (2.8)	11.5 (1.9)*	11.8 (0.4)	12.5 (3.0)	10.1 (3.2)

Higher scores indicate better performance, with a minimum possible score of 1 and a maximum possible score of 19. The asterisk indicates a significant difference in score in comparison to the control group (before Bonferroni correction).

A pre-planned analysis including only the amblyopia and control groups revealed a main effect of group for balance standard scores (*F*_1,41_ = 8.22, *p* = 0.007) and total standard scores (*F*_1,41_ = 4.84, *p* = 0.03). Participants with amblyopia had significantly lower standard scores in these categories compared to controls. *Post hoc* analysis revealed that the amblyopia group performed significantly worse than controls for the walking with heels raised task (*t*_41_ = −2.12, *p* = 0.040) and the coin-slotting task (*t*_41_ = −2.64, *p* = 0.012). However, these differences did not survive a Bonferroni correction for multiple comparisons.

### Relationship between vision tests and motor scores

A linear regression model predicted that BF score (standardized β = −0.09, 95%, *p* = 0.7), type of amblyopia (standardized β = 0.14, 95%, *p* = 0.4), and inter-ocular acuity difference (standardized β = −0.18, 95%, *p* = 0.4) did not significantly predict total motor function scores for the amblyopia group. Exploratory analyses did not reveal any statistically significant associations between any clinical variables and total scores for balance (*F*_3,18_ = 0.89, *p* = 0.45), catching and throwing (*F*_3,18_ = 0.94, *p* = 0.44), or manual dexterity (*F*_3,18_ = 0.70, *p* = 0.57) in the amblyopia group.

## Discussion

Our results demonstrate an association between amblyopia and motor function impairment in children in China. This suggests that cultural factors that influence motor development in China do not offset the impact of amblyopia on motor function. Although it is not possible to test for a causal relationship between amblyopia and impaired motor function in humans, it is possible that the neurodevelopmental deficits that characterize amblyopia directly influence visuomotor control (Webber et al., 2008; Niechwiej-Szwedo et al., 2014; Kiorpes and Daw, 2018).

Patients with amblyopia had worse balance and total motor scores than controls. Zipori et al. (2018) similarly found significantly worse balance scores in patients with strabismic amblyopia compared to controls (assessed using the Bruininks-Oseretsky Test of Motor Proficiency Test). Sa et al. (2021) also reported impairments in the stability and locomotory components of the Motor Competence Assessment Battery in patients with both corrected and non-corrected amblyopia compared to controls. Sa et al. did not observe a deficit for manipulative tasks such as throwing velocity or kicking a ball. However, it was unexpected that manual dexterity was not impaired in our sample of children with amblyopia as this is relatively consistent finding (Webber et al., 2008; Suttle et al., 2011; Kelly et al., 2020). It is possible that cultural influences on motor development influence the association between amblyopia and motor function. Alternatively, a larger sample size may be required to reveal subtle between-group manual dexterity differences.

We did not find that motor function scores in our no-amblyopia BVD group were significantly different from controls, although scores in the no-amblyopia BVD group fell between those of the control and amblyopia groups for most MABC-2 sub-tests. Our results differ from those reported by Kelly et al. (2020) and Caputo et al. (2007) who did observe an association between no-amblyopia BVDs and motor impairment. An inspection of the clinical characteristics revealed that our group had less severe binocular vision deficits (BF score of 2.1 ± 0.8) than the group recruited by Kelly et al. (2020) (BF score of 3.1 ± 1.0) which may explain the difference in results.

We did not observe an association between stereopsis and total motor function scores in our amblyopia group. While our finding that stereopsis did not predict motor scores is consistent with a subset of prior studies in the field (Webber et al., 2008; Zipori et al., 2018; Ibrahimi et al., 2021), others have found a link between stereopsis and motor function in patients with amblyopia.

Methodology may play a role in these diverging findings. Grant et al. (2014) and Suttle et al. (2011) used precise motion tracking devices to investigate the intricacies of motor patterns, which may be sensitive to more subtle differences than standardized tests that generate a score regardless of the motor strategy used to complete the task. A more detailed summary of the literature has already been covered by Niechwiej-Szwedo et al. (2019).

One possible explanation for why we did not see a link between stereopsis and motor function is that our sample included young children who may still have been in a phase of motor development that involves feedforward, ballistic movements that are not corrected in real time by visual feedback (Suttle et al., 2011). Another possibility is the lack of statistical power. A larger sample may allow for further separation of the etiologies, and more information about the no-amblyopia BVD group. For example, patients with strabismus and nil stereopsis had poorer visuo-motor coordination scores on the Developmental Coordination Disorder Questionnaire (Vagge et al., 2021). Ibrahimi et al. (2021) found that patients with exotropic strabismus and nil stereopsis performed worse on the Visual-Motor Integration Test of Beery (6th edition) than those with exotropia and measurable stereopsis, but this effect was not seen in patients with esotropia. There are numerous factors to consider when trying to map any specific, potential causes of motor function deficits in patients with BVDs.

Our results contribute to a growing literature demonstrating that motor deficits are an important component of amblyopia (Birch et al., 2019a). Measures of motor function should be incorporated into clinical trials of existing and novel amblyopia therapies to assess real-world outcomes for patients that extend beyond monocular visual acuity.

## Data availability statement

The raw data supporting the conclusions of this article will be made available by the authors, without undue reservation.

## Ethics statement

The studies involving human participants were reviewed and approved by the University of Waterloo Ethics Review Board and Zhongshan Ophthalmic Centre Ethics Review Board. Written informed consent to participate in this study was provided by the participants’ legal guardian/next of kin.

## Author contributions

All authors listed have made a substantial, direct, and intellectual contribution to the work, and approved it for publication.

## References

[B1] Birch E. E. Castaneda Y. S. Cheng-Patel C. S. Morale S. E. Kelly K. R. Beauchamp C. L. (2019a). Self-perception in children aged 3 to 7 years with amblyopia and its association with deficits in vision and fine motor skills. *JAMA Ophthalmol.* 137 499–506. 10.1001/jamaophthalmol.2018.7075 30763432 PMC6512458

[B2] Birch E. E. Castaneda Y. S. Cheng-Patel C. S. Morale S. E. Kelly K. R. Beauchamp C. L. (2019b). Self-perception of school-aged children with amblyopia and its association with reading speed and motor skills. *JAMA Ophthalmol.* 137 167–174. 10.1001/jamaophthalmol.2018.5527 30452518 PMC6439832

[B3] Birch E. Williams C. Drover J. Fu V. Cheng C. Northstone K. (2008). Randot preschool stereoacuity test: Normative data and validity. *J. AAPOS* 12 23–26. 10.1016/j.jaapos.2007.06.003 17720573 PMC2577836

[B4] Buckley J. G. Panesar G. K. MacLellan M. J. Pacey I. E. Barrett B. T. (2010). Changes to control of adaptive gait in individuals with long-standing reduced stereoacuity. *Invest. Ophthalmol. Vis. Sci.* 51 2487–2495. 10.1167/iovs.09-3858 20335609

[B5] Caputo R. Tinelli F. Bancale A. Campa L. Frosini R. Guzzetta A. (2007). Motor coordination in children with congenital strabismus: Effects of late surgery. *Eur. J. Paediatr. Neurol.* 11 285–291. 10.1016/j.ejpn.2007.02.002 17403610

[B6] Chow S. M. Henderson S. E. Barnett A. L. (2001). The movement assessment battery for children: A comparison of 4-year-old to 6-year-old children from Hong Kong and the United States. *Am. J. Occup. Ther.* 55 55–61. 10.5014/ajot.55.1.55 11216367

[B7] Grant S. Moseley M. J. (2011). Amblyopia and real-world visuomotor tasks. *Strabismus* 19 119–128. 10.3109/09273972.2011.600423 21870915

[B8] Grant S. Suttle C. Melmoth D. R. Conway M. L. Sloper J. J. (2014). Age- and stereovision-dependent eye-hand coordination deficits in children with amblyopia and abnormal binocularity. *Invest. Ophthalmol. Vis. Sci.* 55 5687–57015. 10.1167/iovs.14-14745 25097239 PMC4160093

[B9] Ibrahimi D. Mendiola-Santibanez J. D. Gkaros A. P. (2021). Analysis of the potential impact of strabismus with and without amblyopia on visual-perceptual and visual-motor skills evaluated using TVPS-3 and VMI-6 tests. *J. Optom.* 14 166–175. 10.1016/j.optom.2020.04.002 32535162 PMC8093528

[B10] Ke L. Du W. Wang Y. Duan W. Hua J. Barnett A. L. (2020). The movement ABC-2 test in China: Comparison with UK norms for 3-10 year olds. *Res. Dev. Disabil.* 105:103742. 10.1016/j.ridd.2020.103742 32711248

[B11] Kelly K. R. Jost R. M. De La Cruz A. Birch E. E. (2015). Amblyopic children read more slowly than controls under natural, binocular reading conditions. *J. AAPOS* 19 515–520. 10.1016/j.jaapos.2015.09.002 26610788 PMC4688187

[B12] Kelly K. R. Morale S. E. Beauchamp C. L. Dao L. M. Luu B. A. Birch E. E. (2020). Factors associated with impaired motor skills in strabismic and anisometropic children. *Invest. Ophthalmol. Vis. Sci.* 61:43. 10.1167/iovs.61.10.43 32845292 PMC7452850

[B13] Kiorpes L. Daw N. (2018). Cortical correlates of amblyopia. *Vis. Neurosci.* 35:E016. 10.1017/S0952523817000232 29905122

[B14] Mazyn L. I. Lenoir M. Montagne G. Delaey C. Savelsbergh G. J. (2007). Stereo vision enhances the learning of a catching skill. *Exp. Brain Res.* 179 723–726. 10.1007/s00221-007-0957-5 17487478

[B15] Moke P. S. Turpin A. H. Beck R. W. Holmes J. M. Repka M. X. Birch E. E. (2001). Computerized method of visual acuity testing: Adaptation of the amblyopia treatment study visual acuity testing protocol. *Am. J. Ophthalmol.* 132 903–909. 10.1016/s0002-9394(01)01256-9 11730656

[B16] Niechwiej-Szwedo E. Colpa L. Wong A. M. F. (2019). Visuomotor behaviour in amblyopia: Deficits and compensatory adaptations. *Neural Plast.* 2019:6817839. 10.1155/2019/6817839 31281344 PMC6590572

[B17] Niechwiej-Szwedo E. Goltz H. C. Chandrakumar M. Wong A. M. (2014). Effects of strabismic amblyopia and strabismus without amblyopia on visuomotor behavior: III. Temporal eye-hand coordination during reaching. *Invest. Ophthalmol. Vis. Sci.* 55 7831–7838. 10.1167/iovs.14-15507 25389201

[B18] O’Connor A. R. Birch E. E. Anderson S. Draper H. Group F. R. (2010). The functional significance of stereopsis. *Invest. Ophthalmol. Vis. Sci.* 51 2019–2023. 10.1167/iovs.09-4434 19933184

[B19] Pang A. W. Fong D. T. (2009). Fundamental motor skill proficiency of Hong Kong children aged 6-9 years. *Res. Sports Med.* 17 125–144. 10.1080/15438620902897516 19731174

[B20] Sa C. Luz C. Pombo A. Rodrigues L. P. Cordovil R. (2021). Motor competence in children with and without ambliopia. *Percept. Mot. Skills* 128 746–765. 10.1177/0031512520987359 33435851

[B21] Suttle C. M. Melmoth D. R. Finlay A. L. Sloper J. J. Grant S. (2011). Eye-hand coordination skills in children with and without amblyopia. *Invest. Ophthalmol. Vis. Sci.* 52 1851–1864. 10.1167/iovs.10-6341 21212188 PMC3101694

[B22] Vagge A. Pellegrini M. Lester M. Musolino M. Giannaccare G. Ansaldo R. (2021). Motor skills in children affected by strabismus. *Eye* 35 544–547. 10.1038/s41433-020-0894-0 32350454 PMC8027444

[B23] Webber A. L. Wood J. M. Thompson B. (2016). Fine motor skills of children with amblyopia improve following binocular treatment. *Invest. Ophthalmol. Vis. Sci.* 57 4713–4720. 10.1167/iovs.16-19797 27607417

[B24] Webber A. L. Wood J. M. Gole G. A. Brown B. (2008). The effect of amblyopia on fine motor skills in children. *Invest. Ophthalmol. Vis. Sci.* 49 594–603. 10.1167/iovs.07-0869 18235004

[B25] Webber A. L. Wood J. M. Thompson B. Birch E. E. (2019). From suppression to stereoacuity: A composite binocular function score for clinical research. *Ophthalmic Physiol. Opt.* 39 53–62. 10.1111/opo.12599 30628744 PMC6628341

[B26] Zipori A. B. Colpa L. Wong A. M. F. Cushing S. L. Gordon K. A. (2018). Postural stability and visual impairment: Assessing balance in children with strabismus and amblyopia. *PLoS One* 13:e0205857. 10.1371/journal.pone.0205857 30335817 PMC6193669

